# Enhancing Probiotic Viability in Yogurt: The Role of Apple Fibers in Supporting *Lacticaseibacillus casei* ATCC 393 During Storage and Gastrointestinal Transit

**DOI:** 10.3390/foods14030376

**Published:** 2025-01-24

**Authors:** Dimitra Dimitrellou, Eleni Sakadani, Panagiotis Kandylis

**Affiliations:** Department of Food Science and Technology, Ionian University, GR-28100 Argostoli, Greece; esakadani@ionio.gr

**Keywords:** gastric fluid, intestinal fluid, INFOGEST static protocol, bile salts, functional foods, prebiotic properties, simulated digestion

## Abstract

Probiotics are widely recognized for their health benefits, but their viability during food processing and digestion poses significant challenges. The present study evaluated the impact of incorporating apple fibers into yogurt on the viability of the probiotic strain *Lacticaseibacillus casei* ATCC 393 during production, storage, and simulated gastrointestinal digestion. Apple fibers, a by-product of apple processing, were used as a prebiotic ingredient due to their functional and technological benefits. The incorporation of apple fibers increased probiotic viability during 28 days of refrigerated storage, improving it from 90.4% in the control yogurt to 93.9%. Under simulated gastrointestinal conditions, yogurt alone acted as a protective matrix, preserving probiotic viability, during gastric (71.0% at pH 2 after 3 h) and intestinal digestion (73.3% at 0.3% bile salts after 6 h). The inclusion of apple fibers further enhanced this protection, reducing probiotic viability loss in both gastric (81.9% at pH 2 after 3 h) and intestinal (79.0% at 0.3% bile salts after 6 h) environments. Similar results were obtained using the INFOGEST 2.0 static protocol. After the completion of the protocol (oral, gastric and intestinal phase) a viability of 71.1% (6.61 logCFU/g) was observed in the yogurt with apple fibers compared to 64.5% (6.10 logCFU/g) in the control yogurt. This enhanced protection could be attributed to the potential prebiotic properties of apple fibers, including their pectin and cellulose content, which may shield probiotics from acidic and enzymatic degradation. These findings highlight the potential of apple fiber-enriched yogurt as a functional food that supports probiotic viability during storage and throughout gastrointestinal transit. These insights may open the way for developing new food products with enhanced health benefits, aligning with growing consumer demand for functional foods.

## 1. Introduction

Probiotic foods have been an important part of human history through the tradition of fermented foods. While they are most commonly associated with dairy-based products, non-dairy fermented foods also serve as valuable sources of probiotics [1]. The word probiotic is derived from the Greek language, which means ‘for life’, while in 2001, the Food and Agriculture Organization proposed a useful scientific definition (later grammatically corrected) as “live microorganisms that, when administered in adequate amounts, confer a health benefit on the host” [2]. This definition is accepted without changes today.

The consumption of probiotics has been associated with numerous health benefits related to the prevention and reduction of many diseases, like allergic diseases, obesity, type 1 and type 2 diabetes, cancer, hypercholesterolemia, lactose intolerance, inflammatory bowel disease, diarrhea, irritable bowel syndrome, and brain and central nervous system diseases [3,4]. In order to deliver their health benefits to the host, probiotics should be consumed in sufficient amounts. This amount has not been specified, but the majority of the research works agree that there is a functional dose of probiotics, usually at a level of 10^9^ colony forming units (CFU) per serving or per day according to the regulatory approaches in Canada and Italy, respectively [2]. However, the most challenging issue in probiotics in foods is to maintain these high numbers during food processing, storage, and most importantly, during the stress conditions of the gastrointestinal tract [4].

The food matrix plays a vital role in the maintenance of probiotic viability. Dairy products and especially yogurt are traditional vehicles for the delivery of probiotics. However, in some cases, yogurt characteristics and storage may pose challenges for the viability of these beneficial microorganisms. Some examples include its low pH (4.0–4.6) that may reduce the viability of pH-sensitive probiotics, potential competition of probiotics with yogurt starter microorganisms, and the need for a shelf life, usually up to 28 days.

According to the Global Prebiotic Association, a prebiotic is “a compound or ingredient that is utilized by the microbiota producing a health or performance benefit” [5]. Prebiotics include conjugated linoleic acid, polyunsaturated fatty acid, phenolics, phytochemicals, dietary fibers, and oligosaccharides like fructooligosaccharides, inulin, galactooligosaccharides, mannanoligosaccharide, xylooligosaccharide, and human milk oligosaccharides [6]. Fructooligosaccharides and galactooligosaccharides are the most researched prebiotics, while others like phenolics and phytochemicals are promising candidates. The combination of probiotics and prebiotics results in synbiotic products with numerous health benefits, and therefore, several studies have highlighted their importance, especially in dairy products [7,8,9].

*Lacticaseibacillus casei* ATCC 393 is known for its probiotic properties and its ability to survive in various conditions makes it advantageous for food applications. Therefore, it has been successfully added to various food matrices and mainly dairy products, such as fermented milk [10,11], yogurt [12,13], and cheese [14]. This microorganism has been proven capable of surviving in satisfactory numbers at low pH and simulated gastrointestinal conditions, especially in microencapsulated form [15,16,17,18]. Moreover, it is well known for its potential health benefits like the regulation of intestinal microbiota [19]; tumor-inhibition, pro-apoptotic and anti-proliferative effects [20]; production of bioactive peptides in fermented milk [10]; and the alleviation of intestinal barrier dysfunction [21].

Apple pomace is the by-product of apple processing, primarily generated during the production of apple juice or cider, and it consists of the leftover skins, seeds, cores, and pulp [22]. Several studies have described the incorporation of apple pomace and other apple by-products in yogurts and fermented milks. These studies have demonstrated that apple pomace powder possesses excellent water retention capacity, solubility, and swelling properties, in addition to its ability to increase the viscosity of milk [23]. Furthermore, the produced yogurts presented increased antioxidant activity, dietary fibers, and lower syneresis, with improved textural characteristics, making the structure of the yogurt firmer and more cohesive [24,25]. The addition of apple pomace flour resulted in yogurt with increased total phenolic content and antioxidant activity, while the yogurt extracts inhibited colon cancer cells viability [26]. Finally, the addition of apple peel polyphenol extract increased antioxidant activity and the probiotic viability of yogurts [27].

However, the majority of the above studies evaluated the effect of apple by-products (pomace, extracts, etc.) on the physicochemical, textural and rheological characteristics without in-depth evaluation of their effects on probiotic culture during processing, storage and especially during gastrointestinal conditions. Therefore, the aim of the present study is to fill this gap in the literature by evaluating the viability of probiotic culture during yogurt production, storage and passage through the gastrointestinal tract. Even a slight increase in the number of probiotic *L. casei* cells that survive the gastrointestinal conditions is crucial to guarantee the delivery of their potential health benefits to the host.

## 2. Materials and Methods

### 2.1. Raw Materials

Commercial fresh pasteurized and homogenized cow’s milk (per 100 mL: fat 3.5 g, sugars 4.7 g, proteins 3.2 g; Galpo, Mevgal S.A., Thessaloniki, Greece) and skimmed milk powder (per 100 g: fat 1.0 g, carbohydrates 48.5 g, proteins 34.0 g; Krousarakis Vassilis & SIA OE, Athens, Greece) were used. The apple fibers were a product of organic farming, containing per 100 g: fat 3 g, carbohydrates 35 g, proteins 4 g, dietary fibers 55 g (“Ola Bio”, Bioygeia S.A., Athens, Greece).

### 2.2. Cultures

The thermophilic culture CH-1 Yo-Flex^®^ (Chr. Hansen, Hørsholm, Denmark), a mixture of *Streptococcus thermophilus* and *Lactobacillus delbrueckii* subsp. *bulgaricus*, was used for yogurt production after activation according to the supplier’s instructions. The probiotic strain, *Lacticaseibacillus casei* ATCC 393 (DSMZ, Braunschweig, Germany), was used, which was grown in de Man, Rogosa and Sharpe (MRS, Laboratorios Conda S.A., Madrid, Spain) medium at 37 °C. The culture was re-activated from a stock stored in MRS with glycerol at −18 °C by inoculating into MRS broth and placed at 37 °C for about 18 h, with two successive inoculations (10 and 100 mL). The microorganism was harvested by centrifugation (4000 rpm for 4 min, Universal 320R, Hettich, Tuttlingen, Germany) and then washed with Ringer’s solution (1/4 strength, MERCK KGaA, Darmstadt, Germany) twice (10 mL). The microorganism after the washes was used either to produce the yogurt as it was, or to measure its viability after re-dissolution in distilled water (100 mL).

### 2.3. Yogurt Production and Storage Conditions

Skimmed milk powder (30 g/L) was added to fresh pasteurized cow’s milk, mixed and finally heated for 30 min at 80 °C. Then, the mixture was cooled to 42 °C, inoculated with the culture of *L. casei* ATCC 393 (4 g/L) and after 30 min with the starter culture CH-1 (0.3% *v*/*v*). The mixture was incubated at 42 °C until the pH reached a value of about 4.65. The yogurt was then cooled to 20 °C, stirred gently, dispensed into containers and placed at 4 °C for up to 28 days. Samples were taken after 1, 7, 14, 21, and 28 days of storage. For the preparation of yogurt with apple fibers, the same procedure was followed, but before heating to 80 °C, the fibers were added to the milk (2% *w*/*w*).

### 2.4. Simulated Gastric Fluid

The simulated gastric fluid (SGF) was prepared by adding pepsin (3 g/L) to NaCl solution (5 g/L), the pH value was adjusted to 3 or 2 with 0.1 M HCl solution, and then it was filter sterilized (0.45 µm) [18]. The yogurt sample (1 g) was mixed with 9 mL of SGF, homogenized in a vortex for 10 s and placed in a water bath at 37 °C. After 1 min, 30 min, 60 min, 120 min and 180 min, 1 mL was taken to measure *L. casei* cell viability.

### 2.5. Simulated Small Intestinal Fluid

The simulated small intestinal fluid (SIF) solution consisted of pancreatin (final concentration of 1 g/L in NaCl solution (5 g/L)) with or without the presence of bile salts (3 g/L) [28]. The pH value was adjusted to 7 with 0.1 M NaOH solution. All solutions were filter sterilized (0.45 µm). The yogurt sample (1 g) was mixed with 9 mL of SIF, homogenized in a vortex for 10 s and placed in a water bath at 37 °C. After 1 min, 120 min, 180 min, 240 min and 360 min, 1 mL of sample was taken and the cell viability of *L. casei* was determined.

### 2.6. In Vitro Digestion Using INFOGEST 2.0 Static Protocol

Yogurt samples were studied under in vitro digestion conditions according to the INFOGEST 2.0 protocol [29], with some modifications. Yogurt samples (5 g) were mixed (1:1) with amylase-free saliva simulating fluid (SSF) and vortexed for 2 min. The yogurt/SSF mixture was then mixed (1:1) with simulated gastric fluid (SGF) containing lipase (60 U/mL) and pepsin (2000 U/mL). The pH of the mixture was adjusted to 3 (HCl 1 N) and incubated for 2 h, under agitation at 100 rpm (Labnet ProBlot™ Rocker 25, Edison, NJ, USA), at 37 °C. Then, simulated intestinal fluid (SIF), containing pancreatin (100 U trypsin/mL) and bile salts (10 mM/L), was added (1:1) to the mixture and the pH value was adjusted to 7 (NaOH 1 N). The resulting mixture was incubated for 2 h under agitation at 100 rpm, at 37 °C. At the end of each simulation phase, samples (1 mL) were taken to determine the viability of *L. casei* cells. Also, corresponding samples were taken during the simulated intestinal phase at various time intervals (15, 30, 60, and 120 min).

### 2.7. Determination of L. casei Viability

Yogurt samples (10 g) or samples from simulated gastrointestinal conditions (1 mL) were diluted in sterile ¼ Ringer’s solution and the appropriate serial dilutions were prepared. *L. casei* was cultured using lithium propionate MRS agar (lithium chloride 2 g/L and sodium propionate 3 g/L) at 37 °C [18]. The results are presented as logCFU/g or as viability % using the following equation:

(1) $$viability\;\%=\frac{\log CFU/g\;\left(measured\right)}{\log CFU/g\;\left(initial\right)}\times 100\%$$

### 2.8. Statistical Analysis

All experiments were carried out in triplicate and duplicate samples were collected for each analysis. Data are reported as mean ± standard deviation. Experimental data were evaluated for their significance by analysis of variance (ANOVA) using Tukey’s honest significant difference (HSD) test in SPSS statistics software version 29 (IBM Inc., New York, NY, USA).

## 3. Results and Discussion

### 3.1. Probiotic Survival During Storage

Although fermented milks with fruits and fruit products are very popular in the market, the majority of the available research studies on these fruit-based dairy products has focused on evaluating their effect on textural, flavor and general sensory characteristics, while studies on the viability of the probiotics are limited [30]. The main concern of using fruits in yogurts and fermented milks is their ability to further increase the acidity of the food and therefore make the conditions for probiotic survival harsher. The majority of available studies refer to no significant effects on probiotic viability by the addition of fruit preparations, especially for probiotics like *L. acidophilus*, *L. rhamnosus* GG, and *L. casei*, and in some cases, even an improvement [30]. Probiotics more sensitive in fruit preparations mainly belong to the group of Bifidobacteria [31,32], while a reduction in viability has been reported, in some cases, also for *L. acidophilus* [30,31]. The main factors that negatively affect the viability of probiotics in food matrices are the presence of oxygen, low pH, low temperatures, redox potential, and water activity [33,34].

**Figure 1:**
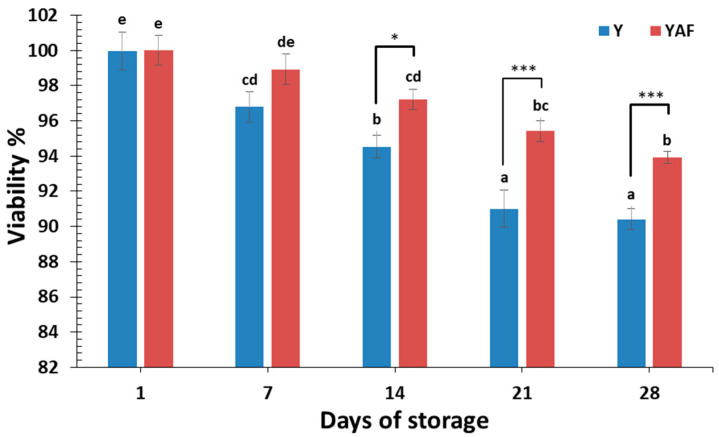
Effect of apple fibers addition on viability of *L. casei* cells during yogurt storage at 4 °C (Y: control yogurt; YAF: yogurt with apple fibers; ^a–e^ Means with different lowercase superscripts differ significantly (*p* < 0.05); Significant different values *: *p* < 0.05; ***: *p* < 0.001).

In the present study, the initial numbers of *L. casei* in yogurt were 9.46 and 9.30 logCFU/g for control yogurt and yogurt with apple fibers, respectively. Therefore, the addition of apple fibers did not affect the initial numbers of probiotic as was also reported with *L. acidophilus* and apple pomace fortified yogurt [35] and yogurt with apple pomace flour [26]. On the other hand, a reduction in the numbers of *B. bifidum* was reported in yogurt with apple pomace flour [26]. During refrigerated storage of yogurt, a significant (*p* < 0.05) reduction in the viability of *L. casei* in both control and apple fiber yogurt was observed (Figure 1). This reduction was more rapid in the case of the control yogurt sample. More specifically, a slight but not significant difference in *L. casei* viability was observed after 7 days of storage. After 14 days, the differences were significant between the control and yogurt with apple fibers (*p* < 0.05) and more significant after 21 days (*p* < 0.001). After 28 days, a viability of 90.4% (8.55 logCFU/g) and 93.9% (8.74 logCFU/g) was reported for control yogurt and yogurt with apple fibers, respectively. The addition of apple fibers seems to promote the viability of probiotics, resulting in a 0.56 logCFU/g reduction compared to 0.90 logCFU/g without apple fibers. In previous studies of our group using the same probiotic strain and conditions, the use of microencapsulation retained the viability of probiotics after 28 days of storage, resulting in a 0.32 logCFU/g reduction in the case of alginate capsules and a 0.75 logCFU/g reduction in the case of spray-dried microcapsules [17,18]. The beneficial effect of apple fibers may be attributed to their role as prebiotics that are metabolized by probiotics and the potential protection of the probiotics from the acidic environment. According to Senadeera et al. [36], probiotics can metabolize fiber and other nutrients from fruit, which may lead to better maintenance of their viability when compared to the control samples. In a similar study, the prebiotic potential of apple peel polyphenol extract in yogurt was proposed since it retained high viability of probiotics, *B. lactis* and *L. acidophilus*, during storage [10]. Previous studies reported the satisfactory viability of *L. casei* ATCC 393 under refrigerated storage of yogurt and fermented milks either in free or encapsulated form [10,13,17,18].

### 3.2. Probiotic Survival During Simulated Gastric Phase and Low pH

One of the most important properties for a microorganism to be classified as a probiotic is its ability to survive in sufficiently high numbers during inoculation, food production, food processing and storage, as well as while passing through the gastrointestinal tract during digestion. Thus, it becomes clear that even if the microorganism is present in high numbers in food, if it cannot survive during digestion, it will not be able to reach the intestine in a viable form, and therefore its functionality is questionable. The low pH and the antimicrobial action of pepsin are the main harmful factors affecting the viability of probiotics in the stomach. For this reason, studying the viability of probiotics under these conditions was deemed necessary.

In this study, the effect of yogurt, with and without apple fibers, on the viability of the probiotic *L. casei* ATCC 393 cells was evaluated for 180 min in conditions simulating gastric juice (at pH levels 3 and 2, in the presence of pepsin). The pH values of 2 and 3 were selected since it is known that the pH of the stomach generally ranges from 2.5 to 3.5 [28].

Several previous studies highlighted the satisfactory viability of *L. casei* ATCC 393 free cells at pH 3, resulting in a reduction of 1.5–1.9 logCFU/g after 2–3 h [18,19]. At pH 2, a more significant drop in viability has been reported of 4.0–5.2 logCFU/g after 2–3 h [15,18,19]. In these studies, a marked decline in viability was observed between 30 to 60 min, with a particularly significant drop after 120 min, resulting in values lower than 4.0 logCFU/g by the end of the storage.

However, in the present study the results highlight the protective effect of yogurt (Table 1). More specifically, the presence of the microorganism in the yogurt matrix, either with or without apple fibers, led to improved results. While a decrease in viability was still observed over time (*p* < 0.05), it was not as significant as that observed for free cells [18]. More specifically, at pH 3, the decline in viability was 0.59 logCFU/g for control yogurt and 0.25 logCFU/g for yogurt with apple fibers. In previous studies of our group using the same probiotic strain and gastric conditions, the use of microencapsulation, without yogurt, retained the viability of probiotics after 3 h at pH 3, resulting in a 0.53 logCFU/g reduction in the case of alginate capsules and a 0.36 logCFU/g reduction in the case of spray-dried microcapsules [17,18]. These results highlight the protective role of the food matrix and especially yogurt on probiotic cells during gastric conditions. A more important decline in the viable numbers of *L. casei* cells was observed in the gastric conditions at pH 2. After 1 h, the viability in control yogurt was 86.5% (8.18 logCFU/g) and in yogurt with apple fibers, 90.8% (8.44 logCFU/g). At the end of the storage, after 3 h, the viability was 71.0% and 81.9% (6.72 and 7.62 logCFU/g) for control yogurt and yogurt with apple fibers, respectively. Comparing the results of the present study with those of previous studies of our group, it is clear that there is a protective role of yogurt on probiotics. More specifically, after 3 h at pH 2, a reduction in viability of 4.03 logCFU/g was observed in the case of free cells, 2.74 logCFU/g in yogurt, 1.68 logCFU/g in yogurt with apple fibers, 1.55 logCFU/g in alginate microencapsulated cells and 1.63 logCFU/g in the case of spray-dried microencapsulated cells of *L. casei* ATCC 393 [17,18]. The findings of the present study are in agreement with other studies that confirmed the positive influence of food matrices, such as whey cheese [37], ice cream and yogurt [28], in maintaining high probiotic viability under simulated gastric fluid conditions, as well as the benefits of encapsulated cells in general [15,17,18,19,38]. This protective effect of yogurt at acidic conditions may be attributed to its buffering capacity, better nutrient availability and fat content [39]. Moreover, milk proteins, especially in cheese matrix, have been proven to enhance the survival of bacterial strains in acidic conditions [40]. Finally, the results of the present study clearly highlight the additional protective effect of apple fibers on probiotics in yogurt at the high acidic conditions of the gastric phase, probably due to their prebiotic characteristics.

### 3.3. Probiotic Survival During Simulated Intestinal Phase

Probiotics, after passing through and surviving the harsh environment of the stomach, are confronted with the equally challenging conditions of the small intestine. Specifically, they are exposed to pancreatin, bile salts and pH values of approximately 7–8. As with stomach conditions, food may affect the viability of probiotics under these conditions. In the present work, conditions simulating small intestinal fluids were studied, both in the absence and presence of bile salts.

The survival of probiotics in the presence of bile salts and in small intestinal conditions in general is considered more critical than the corresponding survival in gastric fluid. This is because new delivery systems and special food matrices may help probiotic strains survive the stomach environment, enhancing their chances of colonization in the intestine [41].

Bile salts act as natural “detergents” assisting the digestion and absorption of hydrophobic food components. Their antimicrobial activity results mainly from this “detergent” property, which disrupts bacterial membranes, while their amphiphilic nature makes them strongly inhibitory to bacteria, thus significantly limiting their survival throughout the gastrointestinal tract [37]. Many studies have reported a negligible effect of pancreatic enzymes (in simulated small intestinal conditions without bile salts) on the viability of lactic acid bacteria in vitro [28,42,43]. This suggests that, compared to bile salts, the effect of pancreatin on probiotic survival is relatively low or negligible.

The incorporation of bile salts (0.3%) into the simulated intestine fluids significantly reduced the viability of *L. casei* cells (Table 2). It is generally a common finding in the literature that the presence of bile salts negatively affects the viability of probiotics, either in free form or even in the case of their incorporation into foods [18,19,38].

In the absence of bile salts, *L. casei* showed high viability; however, a decrease occurred over time (Table 2). Specifically, the duration of exposure particularly affected (*p* < 0.001) the cells in control yogurt (8.75 logCFU/g after 360 min) compared to a less significant effect (*p* < 0.01) on cells in yogurt containing apple fibers (9.04 logCFU/g after 360 min). The positive effect of apple fibers was more evident in the presence of bile salts and especially after exposure for 120 min, where higher rates of viability of *L. casei* in yogurt with apple fibers were observed. More specifically, after 120 min, the viability in control yogurt was 78.9% (7.46 logCFU/g) and in yogurt with apple fibers 82.1% (7.63 logCFU/g). At the end of the storage, after 360 min, the viability was 73.3% and 79.0% (6.94 and 7.35 logCFU/g) for control yogurt and yogurt with apple fibers, respectively. It is important to note that free *L. casei* cells struggle to survive in the presence of bile salts. In general, probiotics have a lot of difficulty surviving under these conditions. Specifically, for the strain *L. casei* ATCC 393, the results in the literature confirm its low viability in the presence of bile salts [18,19], with some studies reporting complete lethality [16].

### 3.4. Probiotic Survival During In Vitro Digestion Using INFOGEST 2.0 Static Protocol

The results from the previous sections, where each phase of the gastrointestinal system was studied separately, were particularly encouraging. However, it is necessary to examine the effect of yogurt, with or without apple fibers, on the viability of *L. casei* in a continuous system without interruptions. This will more effectively simulate the conditions of the gastrointestinal system, incorporating the oral phase, and more importantly, considering the influence of each phase on the next. For this reason, the INFOGEST 2.0 system [29] was chosen, which includes the oral, gastric and intestinal phases.

**Figure 2:**
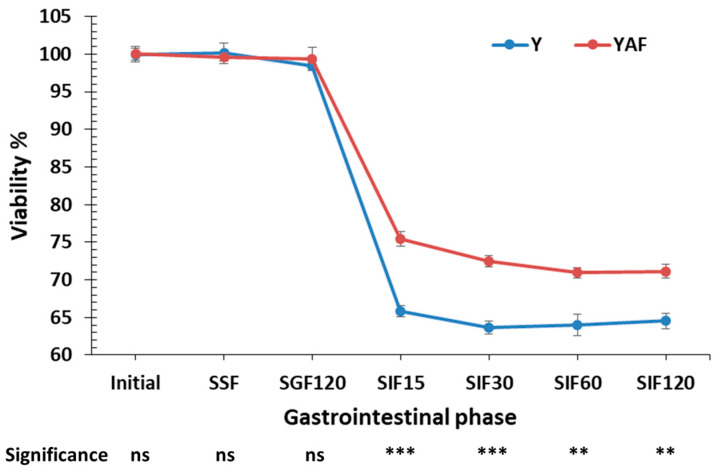
Effect of apple fibers addition on viability of *L. casei* cells in yogurt during in vitro digestion using INFOGEST 2.0 static protocol (Y: control yogurt; YAF: yogurt with apple fibers; SSF: Simulated saliva fluid; SGF: Simulated gastric fluid; SIF: Simulated intestinal fluid; Numbers refer to different time of treatment; Significant different values between Y and YAF in the same phase ns: not significant, *p* > 0.05; **: *p* < 0.01; ***: *p* < 0.001).

The results showed a significant effect of the addition of apple fibers on the viability of *L. casei* cells in the simulated intestinal phase (Figure 2). In all yogurt samples, the effect of the oral and gastric phases was not significant, and the levels of viability of the microorganism remained particularly high, with no significant differences in the presence of apple fibers (in all cases viability > 98%). However, the intestinal phase exhibited a particularly negative effect. Within 15 min of introducing the samples, a decrease in viability was observed by approximately 3.1 logCFU/g for control yogurt (viability of 65.8%) and 2.2 logCFU/g for yogurt with apple fibers (viability of 75.4%). This trend continued throughout the intestinal phase, resulting in a viability of 64.5% (6.10 logCFU/g) and 71.1% (6.61 logCFU/g) at the end of the intestinal phase for control yogurt and yogurt with apple fibers, respectively.

These results are in agreement with observations from the previous sections, where the effect of each phase was studied separately. They also align with a previous study with probiotic lactic acid bacteria in Cheddar cheese [44]. Overall, it can be concluded that the type of food used to incorporate probiotics plays an important role in their survival during passage through the gastrointestinal tract.

The incorporation of apple fibers demonstrated a positive and significant effect on the viability of *L. casei* cells in most cases. In a similar study involving ice cream with probiotic strains, the addition of apple fibers significantly improved survival under gastrointestinal conditions [45]. Apple fibers consist of about 40% soluble pectin, with about half being insoluble cellulose [46]. Pectin and cellulose are less sensitive to chemical agents and demonstrate greater resistance to the gastric environment than other materials that are traditionally used to protect probiotics, such as alginate. These compounds have been found to be suitable mucoadhesive materials [47], which may potentially extend the residence and exposure times under gastrointestinal conditions. Furthermore, in acidic conditions, they appear as aggregates of macromolecules, which are resistant to the proteases and amylases active in the upper gastrointestinal tract. This may explain the enhanced survival of probiotic bacteria in their presence [48]. In general, this protective effect of apple fibers could be attributed to the presence and action of pectin, as numerous studies have reported its prebiotic potential [49] and an increase in the viability of probiotic microorganisms, including *L. casei* ATCC 393, in gastrointestinal conditions when pectin was used as an encapsulation material [50,51,52]. In addition, pectin and cellulose have been characterized as well-known prebiotics, although cellulose is poorly fermented by microorganisms present in human microbiota [53,54]. Another group of compounds that could contribute to the increased viability of probiotics in the present study is the polyphenols present in apple fibers, as was recently highlighted in probiotic yogurt [27]. Furthermore, polyphenols have been recently characterized as potential prebiotics [6]. In our study, in addition to apple pectin, the high carbohydrate (35 g/100 g) and fiber content (55 g/100 g) of apple fibers may be also responsible for the protective effect, especially the carbohydrates since they could be more readily available for utilization by *L. casei* than fibers [55]. However, the prebiotic character of apple fiber is very complicated and may be attributed to a synergistic effect of apple carbohydrates, polysaccharides, polyphenols, pectin and fibers [56].

## 4. Conclusions

Consumer demand for functional foods is continuously increasing, reflecting a broader interest in a healthier diet and lifestyle. Dairy products, and particularly yogurt, enjoy high public acceptance due to their perceived functional characteristics. Although the European Food Safety Authority (EFSA) has not yet recognized the term “probiotics” in food labelling, consumers directly associate it with these products. Nowadays, the food industry is focused on designing and developing products that integrate functional characteristics with probiotics. The incorporation of apple fibers into dairy products has been associated with enhanced functional and technological characteristics such as antioxidant activity, phenolic content, improved texture, and reduced syneresis. The present study specifically evaluated the impact of adding apple fibers on the viability of the probiotic *L. casei* ATCC 393 in yogurt. The results indicated that the addition of apple fibers led to a significant improvement in the viability of probiotics compared to that already provided by the yogurt matrix. The improvement was observed both in the gastric environment and, more significantly, in an intestinal system containing bile salts. These results may open the way for further research into the design of new functional foods that maintain probiotic characteristics during passage through the gastrointestinal system. The present study highlighted the protective effect of apple fibers in probiotics; however, the role of the specific components is unclear. Therefore, future studies should focus on the effect (alone or synergistically) of specific components of apple fibers like carbohydrates, polyphenols, fibers, pectin, etc.

## Figures and Tables

**Table 1:** Effect of apple fibers addition on viability of *L. casei* cells in yogurt under simulated gastric conditions (pH 2 and pH 3).

Time in Simulated Gastric Conditions (min)	Viable *L. casei* Cells (logCFU/g)				Significance (Effect of Apple Fibers)	
	Y (pH 3)	YAF (pH 3)	Y (pH 2)	YAF (pH 2)	pH 3	pH 2
0	9.46 ± 0.10 ^a^	9.30 ± 0.08 ^a^	9.46 ± 0.10 ^a^	9.30 ± 0.08 ^a^	ns	ns
1	8.88 ± 0.02 ^bc^	9.16 ± 0.03 ^ab^	8.65 ± 0.06 ^b^	8.79 ± 0.08 ^b^	***	ns
30	8.97 ± 0.08 ^bc^	8.97 ± 0.06 ^b^	8.42 ± 0.07 ^c^	8.54 ± 0.06 ^c^	ns	ns
60	9.04 ± 0.01 ^b^	9.06 ± 0.11 ^b^	8.18 ± 0.09 ^d^	8.44 ± 0.07 ^cd^	ns	*
120	8.77 ± 0.08 ^bc^	9.00 ± 0.07 ^b^	7.68 ± 0.06 ^e^	8.30 ± 0.10 ^d^	*	***
180	8.87 ± 0.11 ^c^	9.05 ± 0.08 ^b^	6.72 ± 0.08 ^f^	7.62 ± 0.08 ^e^	ns	***
Significance (Effect of Time)	***	**	***	***		

Y: control yogurt; YAF: yogurt with apple fibers; ^a–f^ Means, in the same column, with different lowercase superscripts differ significantly (*p* < 0.05); Significant different values: ns: not significant; *: *p* < 0.05; **: *p* < 0.01; ***: *p* < 0.001.

**Table 2:** Effect of apple fibers addition on viability of *L. casei* cells in yogurt under simulated intestinal conditions (with and without bile salts).

Time in Simulated Intestinal Conditions (min)	Viable *L. casei* Cells (logCFU/g)				Significance (Effect of Apple Fibers)	
	Y	YAF	Y	YAF	Bile Salts	
	Bile Salts 0%		Bile Salts 0.3%		0%	0.3%
0	9.46 ± 0.10 ^a^	9.30 ± 0.08 ^a^	9.46 ± 0.10 ^a^	9.30 ± 0.08 ^a^	ns	ns
1	9.06 ± 0.09 ^b^	9.14 ± 0.06 ^ab^	8.65 ± 0.04 ^b^	8.55 ± 0.08 ^b^	ns	ns
120	8.89 ± 0.08 ^bc^	9.19 ± 0.05 ^ab^	7.46 ± 0.10 ^c^	7.63 ± 0.02 ^c^	**	*
180	8.96 ± 0.06 ^bc^	9.11 ± 0.09 ^b^	7.10 ± 0.06 ^d^	7.47 ± 0.08 ^cd^	ns	**
240	8.85 ± 0.08 ^c^	8.93 ± 0.04 ^c^	7.05 ± 0.07 ^d^	7.42 ± 0.05 ^d^	ns	**
360	8.75 ± 0.02 ^c^	9.04 ± 0.04 ^bc^	6.94 ± 0.03 ^d^	7.35 ± 0.05 ^d^	***	***
Significance (Effect of Time)	***	***	***	***		

Y: control yogurt; YAF: yogurt with apple fibers; ^a–d^ Means, in the same column, with different lowercase superscripts differ significantly (*p* < 0.05); Significant different values: ns: not significant; *: *p* < 0.05; **: *p* < 0.01; ***: *p* < 0.001.

## Data Availability

The original contributions presented in this study are included in the article. Further inquiries can be directed to the corresponding authors.

## Abbreviations

The following abbreviations are used in this manuscript:

ATCC	American Type Culture Collection
CFU	Colony forming unit
*L. casei*	*Lacticaseibacillus casei*
SGF	Simulated gastric fluid
SIF	Simulated intestinal fluid
SSF	Simulated saliva fluid
Y	Control yogurt without apple fibers
YAF	Yogurt with apple fibers
